# Complete Genome Sequences of Six *Legionella pneumophila* Isolates from Two Collocated Outbreaks of Legionnaires’ Disease in 2005 and 2008 in Sarpsborg/Fredrikstad, Norway

**DOI:** 10.1128/genomeA.01367-16

**Published:** 2016-12-15

**Authors:** Marius Dybwad, Tone Aarskaug, Else-Marie Fykse, Elisabeth Henie Madslien, Janet Martha Blatny

**Affiliations:** Norwegian Defence Research Establishment FFI, Kjeller, Norway

## Abstract

Here, we report the complete genome sequences of *Legionella pneumophila* isolates from two collocated outbreaks of Legionnaires’ disease in 2005 and 2008 in Sarpsborg/Fredrikstad, Norway. One clinical and two environmental isolates were sequenced from each outbreak. The genome of all six isolates consisted of a 3.36 Mb-chromosome, while the 2005 genomes featured an additional 68 kb-episome sharing high sequence similarity with the *L. pneumophila* Lens plasmid. All six genomes contained multiple mobile genetic elements including novel combinations of type-IVA secretion systems. A comparative genomics study will be launched to resolve the genetic relationship between the *L. pneumophila* isolates.

## GENOME ANNOUNCEMENT

*Legionella pneumophila* is an opportunistic bacterial pathogen capable of airborne transmission from contaminated freshwater systems to susceptible humans, resulting in a severe pneumonia known as Legionnaires’ disease (1). Sequence-based typing is the standard subtyping method for *L. pneumophila* (2), however, recent cost reductions and increased availability have enabled whole-genome sequencing-based subtyping (3).

Here, we report complete genome sequences of *L. pneumophila* isolates from two collocated outbreaks of Legionnaires’ disease in 2005 and 2008 in Sarpsborg/Fredrikstad, Norway (Table 1). One clinical and two environmental isolates were sequenced from each outbreak. Additional isolate information has been reported elsewhere (4–7).

**TABLE 1:** Isolate information and key genomic features of the *Legionella pneumophila* isolates subjected to whole-genome sequencing in this work

Strain/isolate	Source	Country	Yr	SG^b^	ST^c^	Genome size (bp)	Chromosome accession no.	Chromosome size (bp)	Episome accession no.	Episome size (bp)
FFI102	Clinical	Norway	2005	SG1	ST15	3,431,799	CP016868	3,363,654	CP016869	68,145
FFI103	Environmental	Norway	2005	SG1	ST15	3,431,761	CP016870	3,363,616	CP016871	68,145
FFI329	Environmental	Norway	2005	SG1	ST15	3,431,804	CP016874	3,363,658	CP016875	68,146
FFI104	Clinical	Norway	2008	SG1	ST462	3,362,494	CP016872	3,362,494	—^d^	—
FFI105	Environmental	Norway	2008	SG1	ST462	3,363,998	CP016873	3,363,998	—	—
FFI337	Environmental	Norway	2008	SG1	ST462	3,362,463	CP016876	3,362,463	—	—
Lens^a^	Clinical	France	2004	SG1	ST15	3,405,519	CR628337	3,345,687	CR628339	59,832

a The genome sequence of *L. pneumophila* Lens was obtained from GenBank and included as a reference because sequence-based typing had previously showed that the 2005 isolates had the same ST as Lens (ST15) while the 2008 isolates had a different ST (ST462) but were still closely related to Lens.

b SG, serotype/serogroup.

c ST, sequence type.

d —, not applicable.

Isolates were grown on buffered-charcoal-yeast-extract agar (72 h, 37°C). DNA was purified using Genomic-Tip 100/G (Qiagen, Hilden, Germany). Sequencing was done with PacBio RSII (Menlo Park, CA) and Illumina MiSeq (San Diego, CA). RSII library was prepared using the 20 kb-protocol and size selection done with BluePippin (9 kb-cutoff). Sequencing was done using P6-C4 chemistry and one single-molecule real-time (SMRT) cell. MiSeq library (300 bp paired-end) was prepared with TruSeq PCR-free protocol. Approximately 90,000 RSII and 3,000,000 MiSeq reads were generated for each isolate. RSII reads were *de novo*-assembled with HGAP_v3.0. Minimus2 (AMOS_v3.1) was used for circularization and RS_Resequencing for mapping of RSII reads. MiSeq reads were mapped onto the final RSII assembly with Bionumerics_v7.6 (Applied Maths, Sint-Martens-Latem, Belgium). Annotation was done with NCBI PGAP_v3.3.

All genomes consisted of a 3.36 Mb-chromosome, while the 2005 genomes featured an additional 68 kb—episome showing high similarity (>97%) with a 39 kb-region of the 60 kb-episome of *L. pneumophila* Lens (Table 1). Average coverage was 284× (RSII) and 466× (MiSeq). All genomes showed conserved syntheny with other *L. pneumophila* genomes and highest degree of similarity with Lens (>95%), in agreement with previous sequence-based typing (6). Average G+C content was 38.5% and number of protein-coding genes 2,900, both comparable to Lens (38.4% and 2,932 genes, respectively) (8). *L. pneumophila* often have a dynamic accessory genome consisting of mobile genetic elements, including integrative conjugative elements encoding type-IVA secretion systems (T4ASS), that may facilitate horizontal gene transfer, genome plasticity, and environmental adaption potential (3, 9). All genomes contained Dot/Icm type-IVB secretion system (T4BSS) and genomic island-associated T4ASS (GI-T4ASS). None of the genomes contained Lvh T4ASS, which is present in Lens (3), while all genomes contained Trb (P-type) T4ASS, which is absent in Lens. The 2008 genomes contained a Trb similar to the one in Corby/Alcoy, while the 2005 genomes contained a Trb similar to the one in Lorraine (3). Only the 2005 genomes contained Tra (F-type) T4ASS, which also is present in Lens. Tra was same as in Lens located on the episome in the 2005 genomes. All genomes contained additional virulence-associated elements including RtxA (10) and clustered regularly interspaced short palindromic repeats (CRISPR)-associated systems (Cas) (11). The RtxA in the 2005 genomes shared high similarity (>92%) with the one in Lens, while the RtxA in the 2008 genomes was more similar to the one in Corby/Alcoy.

Comparative genomics will be used to resolve the genetic relationship between the sequenced isolates. Efforts to increase the availability of *L. pneumophila* genomes may serve as an important catalyst of advancements in this field.

### Accession number(s).

This whole-genome sequencing (WGS) project was deposited in GenBank under the accession numbers listed in Table 1.
